# COVID-19 Vaccine Hesitancy: The Perils of Peddling Science by Social Media and the Lay Press

**DOI:** 10.3390/vaccines10071059

**Published:** 2022-06-30

**Authors:** Shabeer Ali Thorakkattil, Suhaj Abdulsalim, Mohammed Salim Karattuthodi, Mazhuvanchery Kesavan Unnikrishnan, Muhammed Rashid, Girish Thunga

**Affiliations:** 1 Pharmacy Services Department, Johns Hopkins Aramco Healthcare (JHAH), Dhahran 34465, Saudi Arabia; shabeer.thorakkattil@jhah.com; 2 Department of Pharmacy Practice, Unaizah College of Pharmacy, Qassim University, Buraydah 52571, Saudi Arabia; 3 Department of Pharmacy Practice, Manipal College of Pharmaceutical Sciences, Manipal 576104, India; salim.kt@manipal.edu (M.S.K.); muhammed.rashid@learner.manipal.edu (M.R.); girish.thunga@manipal.edu (G.T.); 4 Department of Pharmacy Practice, Nitte Gulabi Shetty Memorial Institute of Pharmaceutical Sciences, Mangalore 75018, India; unnikrishnan.mk@nitte.edu.in

**Keywords:** vaccine hesitancy, COVID-19, AEFI, COVID-19 vaccination, vaccine acceptance, medical literature

## Abstract

Introduction: Vaccines are the best tools to end the pandemic, and their public acceptance is crucial in achieving herd immunity. Despite global efforts to increase access to vaccination, the World Health Organization explicitly lists vaccination hesitancy (VH) as a significant threat. Despite robust safety reports from regulatory authorities and public health advisories, a substantial proportion of the community remains obsessed with the hazards of vaccination. This calls for identifying and eliminating possible causative elements, among which this study investigates the inappropriate dissemination of medical literature concerning COVID-19 and adverse events following immunization (AEFI), its influence on promoting VH, and proposals for overcoming this problem in the future. Methods: We searched PubMed, Embase, and Scopus databases, using the keywords “adverse events following immunization (AEFI)”, “COVID-19”, “vaccines” and “hesitancy” and related medical and subjective headings (MeSH) up to 31 March 2022, and extracted studies relevant to the COVID-19 AEFI and associated VH. Finally, 47 articles were chosen to generate a narrative synthesis. Results: The databases depicted a steep rise in publications on COVID-19 AEFI and COVID-19 VH from January 2021 onwards. The articles depicted multiple events of mild AEFIs without fatal events in recipients. While documenting AEFIs is praiseworthy, publishing such reports without prior expert surveillance can exaggerate public apprehension and inappropriately fuel VH. VH is a deep-rooted phenomenon, but it is difficult to zero in on the exact reason for it. Spreading rumors/misinformation on COVID-19 vaccines might be an important provocation for VH, which includes indiscriminately reporting AEFI on a massive scale. While a number of reported AEFIs fall within the acceptable limits in the course of extensive COVID-19 vaccinations, it is important to critically evaluate and moderate the reporting and dissemination of AEFI in order to allay panic. Conclusions: Vaccination programs are necessary to end any pandemic, and VH may be attributed to multiple reasons. VH may be assuaged by initiating educational programs on the importance of vaccination, raising public awareness and monitoring the inappropriate dissemination of misleading information. Government-initiated strategies can potentially restrict random AEFI reports from lay epidemiologists and healthcare practitioners.

## 1. Introduction

“The most important ingredient in all vaccines is trust” [1], and their public acceptance is critical to ending any pandemic [2]. A return to pre-pandemic status is normally possible with an effective vaccine strategy that is successfully implemented throughout all nations [3]. The World Health Organization (WHO) reports say that vaccinations prevented no less than 10 million deaths, globally, between 2010 and 2015 [4]. Despite governments’ official recommendation to get vaccinated, [5] skepticism towards COVID-19 immunization is conspicuously increasing [6,7]. Being new to the world and spreading rapidly, COVID-19 persists despite established preventive measures. Multiple clinical trials for developing and enhancing the quality of vaccines are being undertaken at an unprecedented pace. Nevertheless, the extensive rollout of multiple vaccines in quick succession surprised the public, raising concerns about their efficacy and safety, potentially aggravated by suspected regulatory leniency under political pressure. This might have prompted COVID-19 vaccine hesitancy (VH), a major concern today. Although VH is not new, its reasons being multifactorial, any aspect leading to VH should be seriously considered, especially during the pandemic. Moreover, achieving herd immunity requires both effective vaccines and successful immunization programs [8]. On the other hand, adverse events are quite common following an immunization. However, the manner in which this is explained to the public should endeavor to minimize VH.

Acceptance of any vaccine depends on individual knowledge and prevailing public opinion, wherein published articles contribute substantially. Although peer review avows the quality and reliability of articles, there are many papers available online that can potentially dissuade people from the COVID-19 vaccine. Moreover, the COVID-19 era has witnessed an unprecedented deluge in scientific publishing. Un-authenticated reports on the vaccine have the potential to derail perceptions and promote inappropriate vaccine preferences. Articles should promote the adoption of rational vaccinations that lead to herd immunity, and publications should proactively bring down VH.

This article aims to critically evaluate the mass reporting of COVID-19 AEFIs and formulate our expert opinion on the link between VH and the exaggerated publicity given to publications on AEFIs. We have also outlined the current status of VH, how inappropriate and irresponsible dissemination of distorted peer-reviewed scientific literature aggravates VH, and how this problem could be overcome.

## 2. Methodology

An extensive search was performed on leading databases such as PubMed, Scopus, and Embase by using the keywords “adverse events following immunization (AEFI),” “COVID-19”, “vaccines,” and “hesitancy”, and related medical and subjective headings (MeSH) up to 31 March 2022. A snowball search in Google and Google Scholar identified additional articles related to the topic. Finally, a total of 47 articles were chosen to generate a narrative synthesis linking VH and exaggerated reports of AEFIs. Meanwhile, to visualize the trend concerning publications on COVID-19, VH and its AEFIs, we documented the frequency of the available literature in PubMed from January 2018 to March 2022 in an Excel sheet and employed descriptive statistics presented in graphical format.

## 3. Results

**Figure 1:**
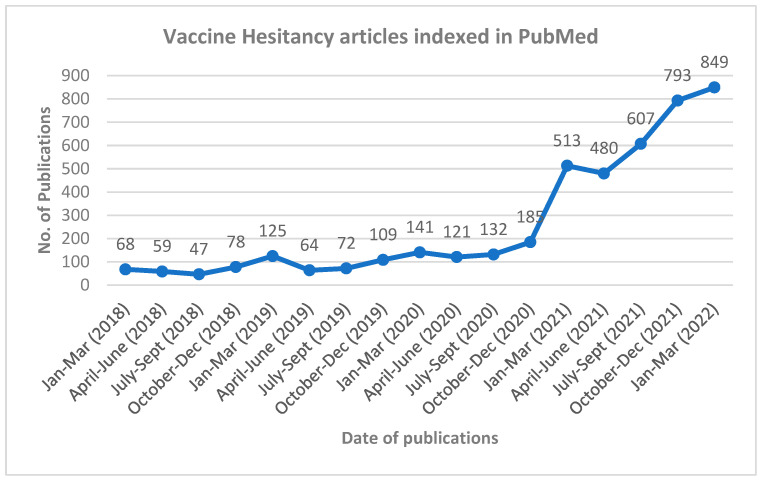
Publication trend on vaccine hesitancy in PubMed.

**Figure 2:**
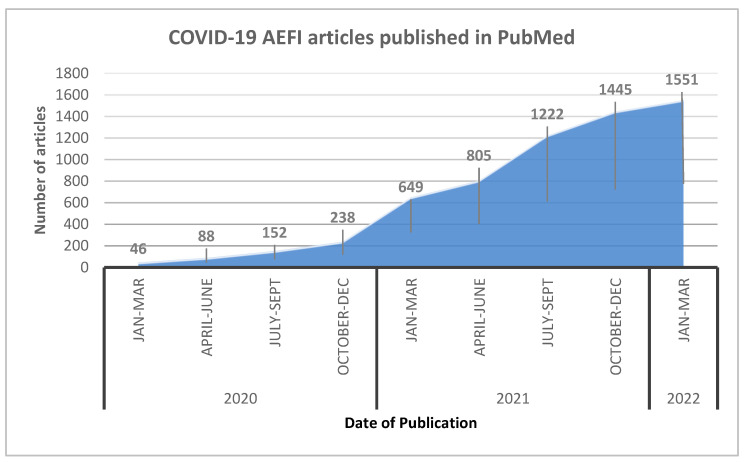
The COVID-19 AEFI papers published in PubMed from 1 January 2020 to 31 March 2022.

**Figure 3:**
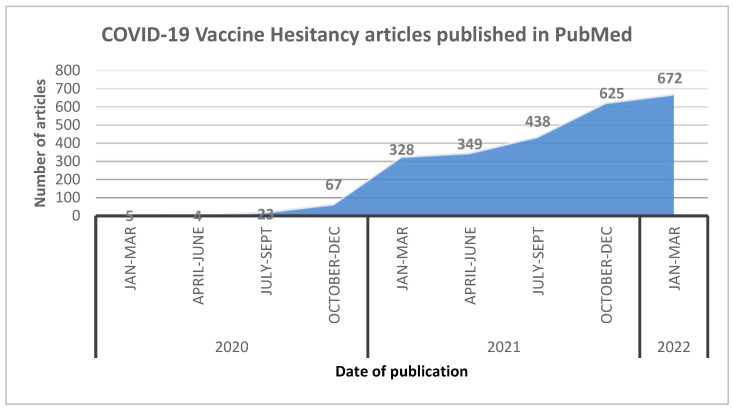
The COVID-19 vaccine hesitancy papers published in PubMed from 1st January 2020 to 31st March 2022.

We conducted an extensive literature search in PubMed using the keywords restricted to the topic. Publication trends over time scale demonstrated a steep rise in the number of publications in three search categories (namely “general vaccine hesitancy”, “COVID-19 vaccine hesitancy” and “COVID-19 AEFI”) during the COVID-19 pandemic compared to before it. A total of 54 relevant articles were considered to generate the narrative synthesis. The number of studies identified through the PubMed search were used to present these data, as illustrated in Figure 1, Figure 2 and Figure 3.

### Vaccine Hesitancy and AEFI Literatures

A PubMed search on VH yielded a good number of papers, as depicted in Figure 1. The pre-COVID-19 vaccine era (January 2018–December 2019) yielded a limited number of VH publications with a yearly average of 25.92 ± 11.03 articles. On the other hand, COVID-19 vaccine launches aggravated public concerns, provoking a steep rise in VH articles from January 2020, with a yearly average of 123.83 ± 90.21 publications. An unpaired *t*-test on the number of published VH papers before and during the COVID-19 pandemic revealed a statistical significance (*p* < 0.001). To be more specific, the PubMed database witnessed a surge in published articles on VH, suggesting that causing alarm in a specific member of a given group can precipitously raise public anxiety among other members of the same group. Thus, COVID-19 vaccines triggered VH towards immunization in general, directed at other available vaccines too [9].

To understand the situation further, we performed a specific search restricted to COVID-19 vaccine AEFIs in PubMed database between 1 January 2020 and 31 March 2022 (Figure 2). Publications on AEFIs increased immensely from its lowest in January-March 2020 (*n* = 46) to its highest after 2 years (*n* = 1551). Cumulatively, 6195 articles on COVID-19 AEFIs were published during this period. Simultaneously, a much more significant contribution from the domain of COVID-19 VH (*n* = 7366) was also observed.

Over time, PubMed-indexed articles showed a rapid increase in COVID-19 VH (Figure 3) and its AEFI-related publications. Surprisingly, all attempts to increase vaccination rates have been neutralized by rising trends in VH.

## 4. Discussion

The concept of VH is multidimensional, and include circumstances such as historical backdrop, trust, geographical location, political situation and agenda, satisfaction, convenience, accessibility and confidence in vaccines. VH has grown with the penetration of technology and is currently at its peak on account of the inappropriate dissemination of information and the spread of misguided or misinterpreted information. AEFIs, which are expected to occur in a minority, are a topic of discussion. Inappropriate dissemination of misleading information by non-scientific self-styled ‘spokespersons’ and social media have promoted COVID-19 VH across the world, especially in developing countries. Here, authors have explored these aspects of COVID-19 VH in view of the available literature and came up with recommendations to overcome VH in the best possible way.

### 4.1. COVID-19 Vaccine Hesitancy

In the context of global efforts to increase access to vaccination, [10] the WHO explicitly mentions that the current threat is VH, defined as a reluctance to vaccinate, despite the availability of vaccines [11]. Social media platforms spread confusing, misleading and unfounded rumors about vaccines even before vaccines were available for administration [12]. Subsequently, frequent mutations in the viral genome and the emergence of new variants augmented the uncertainty [13]. The daily progression of Omicron roused people’s apprehension even among the most vaccine-confident populations [14]. Previous experiences with vaccine-preventable diseases have clearly demonstrated that VH lowers vaccine uptake and increases transmission [15,16]. COVID-19 VH was maximum among those with existing comorbidities, probably driven by reports similar to those by Kaur et al., which cautioned against administering COVID-19 vaccines in females and those with co-morbidities. Additionally, a large multi-centered survey among rheumatology patients from 102 countries (*n* = 7005) suggested that VH was mainly associated with a fear of side effects [17]. Recent articles claimed that COVID-19 vaccines protect individuals from severe infections for up to a period of 9 months, and the immunity may wane within a few months [18,19]. Even though booster doses are highly desirable, recommending multiple shots creates a troublesome dilemma regarding the protection window of COVID-19 vaccines. In this context, inappropriately communicated AEFIs can cultivate a pessimistic attitude towards vaccines and can generate diehard VH.

A survey-based study covering 12 countries, including the USA, Russia, and ten low- and middle-income countries (LMIC), reported that COVID-19 vaccines’ side effects promote hesitancy. Surprisingly, vaccine acceptance among LMICs is higher than that of the USA and Russia [20]. This is supported by a Malaysian study that demonstrated residents’ willingness to get vaccinated [21]. Reports suggest that countries with high literacy rates and financial prosperity have demonstrated greater VH in this context. This might have been facilitated by their urge to search for facts before opting for a system, coupled with easy access to authentic resources. Consequently, VH in developed countries may be a result of the inappropriate dissemination of the scientific literature about AEFI.

Healthcare professionals should be role models for receiving a COVD-19 vaccine. Paradoxically, healthcare workers themselves contribute significantly to VH. The first COVID-19 VH study among US medical students (*n* = 168) at the beginning of the pandemic reported that 23% were hesitant to take the vaccine immediately after FDA approval, due to their concern about side effects [22]. Another study on US medical (*n* = 167) and dental students (*n* = 248) reported a VH of 23% and 45%, respectively [23]. Likewise, a study among Indian medical students (*n* = 1068) reported a VH of 10.3% among the respondents [24]. Another study on healthcare professionals (*n* = 343) reported 44.9% VH towards COVID-19 vaccines [25]. As many as 23.1% of healthcare workers in France (*n* = 1965) expressed VH (at the beginning of the COVID-19 vaccine campaign), of whom 3.9% opposed the COVID-19 vaccine [26]. Awareness about AEFIs can also increase VH’s occurrence. The significant level of VH reported among medical students and healthcare professionals strengthens our arguments that many AEFI publications have contributed to amplifying safety concerns and, eventually, VH.

The rate and intensity of VH can vary for multiple reasons. A study on potentially immunocompromised cancer patients (*n* = 2158) in Eastern China by Jing Hong et al. reported a VH of 24.05%, a vaccination level of 35.54%, and 40.01% willing to get vaccinated. Doubts regarding possible vaccination-related interference with cancer prognosis and safety concerns relating to COVID-19 vaccines were the highlighted worries among participants [27]. Likewise, a study from India (*n* = 803) by Sovan S et al. found that 12.08% of participants considered the vaccination unnecessary for preventing COVID-19. Lack of awareness and knowledge about the COVID-19 pandemic had contributed to the public’s VH [28]. A similar study from Bangladesh (*n* = 591) by Mahmud S et al. demonstrated that 61.16% were willing to take COVID-19 vaccination, out of whom 64.86% wanted to postpone vaccination until safety and efficacy were confirmed. Income, education, age, sex, severity and risk of infection were found to influence their VH [29]. A university-based study (*n* = 614) from the UAE by Jairoun et al. on knowledge, attitude, and the determents of third COVID-19 vaccine booster dose acceptance, reported an average knowledge score of 44.6% and an attitude score of 70.2%. This study also highlighted the importance of developing an educational framework to increase awareness about the importance of vaccination against COVID-19 [30]. Moreover, a systematic review encompassing 209 global studies found that VH was aggravated by a negative perception of vaccine efficacy, safety, convenience, and price, in addition to socio-demographic factors [31]. A study on the elderly population in Italy showed that even educated individuals with a good level of knowledge about the nature of the pathogen, the mode of COVID-19 spread, risk factors, etc., needed effective warnings and messages from public health spokesmen. Paradoxically, even among senior citizens with good knowledge, the pandemic actually promoted unhealthy habits such as smoking. Knowledge alone does not create conducive attitudes automatically. Likewise, awareness about the benefits of vaccination may not automatically improve vaccine compliance in the community. Active and persistent persuasion is often necessary to overcome VH [32]. On the other hand, a study in Japan suggested that willingness to get vaccinated is associated with a strong belief in vaccine effectiveness, and anxiety is associated with an unwillingness to receive the vaccine. Leveraging the psychological support from such populations would enhance the effectiveness of vaccination programs [33].

Baseless rumors constitute a major reason for VH. The spread of misinformation such as vaccine-associated infertility was among the prominent reasons for VH [34]. Another study reported COVID-19 vaccine conspiracy theories circulating through leading social media platforms. Out of 637 COVID-19 vaccine-related statements identified, only 5% were true. The remaining 95% were either false, misleading, or exaggerated [35].

In a global effort to end the COVID-19 pandemic, governments worldwide have enforced mandatory vaccination for international travel, entering workplaces, offices, and shopping malls. Despite social pressure and community restrictions directed against the unvaccinated, VH seems to rise, as reflected by a massive increase in VH publications.

### 4.2. Adverse Events following COVID-19 Vaccination

Adverse events following immunization (AEFI) are quite common and need to be investigated during and after vaccination. Many countries neither have well-defined surveillance and reporting systems nor trained professionals to detect AEFIs, assess causality, manage AEFIs, and eliminate confounders [36,37]. Hoeve et al. identified 154 potentially biased individual case reports on AEFIs that cannot be directly attributed to vaccines but could have occurred from non-compliance to recommended immunization schedules [38]. Healthcare professionals specialized in this discipline have a significant role in separating and resolving confounders from genuine AEFIs. This is imperative because flawed reports would raise VH.

We then surveyed the peer-reviewed literature on the safety and AEFIs of COVID-19 vaccines. An interim analysis of an Indian population revealed 40% AEFIs after the first dose and 15% after the subsequent dose [39]. Pain at the injection site, fever, and headache were the most common mild AEFIs reported, mostly resolved with a single dose of analgesic and antipyretic [40,41]. Another study also featured mild AEFIs that resemble those of non-COVID-19 vaccines [42]. On the other hand, while a few presume that the absence of AEFIs implies a lack of benefit, the majority believe that high doses of vaccines promote AEFIs. Multiple misconceptions on VH prevail.

The WHO recommends that all nations should investigate serious AEFIs [43]. Each AEFI is rated differently depending on vaccine type. A systematic review by Qianhui et al. demonstrated a significantly lower number of AEFIs among inactivated vaccines (local, 23.7%, systemic, 21.0%), protein subunit vaccines (local, 33.0%, systemic, 22.3%), and DNA vaccines (local, 39.5%, systemic, 29.3%), compared to RNA vaccines (local, 89.4%, systemic, 83.3%), non-replicating vector vaccines (local, 55.9%, systemic, 66.3%), and virus-like particle vaccines (local, 100.0%, systemic, 78.9%) [44]. Additionally, a systematic review by Nie et al. cited a few cases of Guillain-Barre syndrome and convulsions following COVID-19 vaccination [45]. In Hong Kong, twenty-eight Bell’s palsy cases were reported with the inactivated vaccine (*n* = 451,939), and 16 from the mRNA vaccine (*n* = 537,205) [46]. A retrospective study conducted in a Swedish population revealed two hundred and seventy-seven vaccinated candidates who were either hospitalized or died because of a recombinant vaccine, mRNA-based vaccine, or nucleoside-modified RNA vaccine [18]. The presence of antibodies against platelet factor 4 or anti-platelet factor 4 was postulated as the reason behind the death [47]. Moreover, clinical and diagnostic parameters indicated thrombosis or thrombocytopenia following cerebral venous thrombosis [48]. Additionally, Althaus et al. recorded eight cases of cerebral hemorrhage, bilateral thromboembolism and renal thrombi following vaccination with recombinant vaccines [49]. However, another report demonstrated that vaccine-induced fatality is extremely low (Laos and Singapore had about 155 AEFIs per million) [50].

In addition, numerous case reports concerning COVID-19 are being circulated online. Many provide useful data to healthcare providers in matters of rare clinical manifestations, diagnostic aspects, and clinical opinions. On the other hand, substandard, predatory journals disseminate misleading information. Reports on AEFIs featured in magazines without peer review or editorial supervision may negatively influence people’s views on the vaccine [51]. This calls for rigorous centralized rules to filter out and regulate such publications [52,53].

Online surveys, a popular research tool, became more popular during the pandemic [54]. Survey-based AEFI studies can be misleading on account of the faulty allocation of subjects and changes in their attitude over time [55]. Opposing views and perceptions of medical professionals can also influence the public’s opinion adversely [56]. Considering the potential impact of AEFI reports on VH, the margin of error should be negligible. For greater authenticity and reliability, active surveillance programs should scrutinize reports on AEFIs (of COVID-19 vaccines) and assess causality to rule out confounders.

Another major problem is that being aware of adverse events per se will increase the odds of experiencing them [57]. For instance, when patients were told that a certain medicine for prostatitis would increase sexual dysfunction, 28.3% more patients actually experienced sexual dysfunction than those who were not informed. Similarly, patients taking medicines for angina pectoris, when warned about possible gastrointestinal side effects, experienced a six-fold increase in gastrointestinal side effects [58]. On the other hand, another interesting study demonstrated the value in redefining adverse events as a sign of a therapeutic effect. When patients (undergoing oral immunotherapy for peanut allergies) were told that side effects were a sign of the treatment actually working, not only were there fewer complaints from patients, but also more reports of better treatment outcomes, including an increase in the level of biomarkers indicating therapeutic success [59]. Pharmacists trained in pharmacovigilance could deploy the above strategy in improving compliance to vaccination and allaying fears about AEFIs.

It follows that articles on AEFIs need to be monitored for content. Mass AE reporting post-COVID-19 vaccination would certainly escalate VH. Figure 2 and Figure 3 clearly depict the rise in articles related to COVID-19 AEFIs, along with the parallel rise in reports concerning VH.

Social networks such as Twitter, Facebook, etc., have hastened information sharing and information cascades (where the same information is spread independently by another) on an immense scale. The prestigious journal *Science* published a report of a large-scale empirical investigation on how social networks encourage the spread of misinformation over truth. Interestingly, a falsehood spreads significantly farther, faster, deeper, and more broadly than a truth. False cascades went deeper and among them, the top falsehoods diffused many levels deeper into the ‘Twittersphere’. Even more interestingly, while the truth rarely spreads beyond 1000 members, the top 1% of false-news cascades spread to as many as 100,000 members. The distribution exhibited a ‘viral branching’ pattern of peer-to-peer diffusion. The truth also took six times longer to reach 1500 members than falsehoods, while a falsehood was 70% more likely to be retweeted. A falsehood was also found to be treated as more novel, receiving greater individual attention and further community diffusion [60].

Sharing unverified AEFI data on social media platforms sets the foundation of lay epidemiology. Such lay epidemiology also shares many features of false news that possess many characteristics of novelty. More importantly, survival instinct prioritizes the avoidance of danger, making humans evolutionarily predisposed to pay greater attention to the threats of AEFIs than the benefits of vaccines. In other words, modern democracies are socially and behaviorally prone to adopt VH.

Politically speaking, social media has the potential to aggravate VH, especially in the liberal democracies of the West, where authority is poorly respected. As understanding peer-reviewed reports of AEFI is the privilege of a tiny circle of experts, what generally reaches the lay public through social media is bound to be a distortion of reality. Since falsehoods and threatening news tend to disseminate more assertively, it is only natural that social media aggravates VH by spreading fear about vaccines and challenging the advice of authorities.

### 4.3. Recommendations to Overcome VH

Effectiveness studies of vaccines in real-world settings play a vital role in informing policy decisions that monitor impact and determine future strategies for vaccination. Health officials should develop extensive educational campaigns addressing AEFI. Furthermore, they should be trained to make strong persuasive recommendations about vaccination, if required, quoting personal experiences. AEFIs discussed between members of small intimate groups of friends, colleagues, and family can spread rapidly, through social media, to the community, even entire populations, generating ‘informed opinions’ that constitute a part of what experts call ‘lay epidemiology’ [61]. The social media companies can review the contents posted on COVID-19 vaccines and consult health authorities to appraise the veracity of such information [62]. The public should be educated about “What resources can be trusted before making a decision?” and motivated to follow instructions only from national authorities and government agencies.

When group behavior is overwhelmingly opposed to a given vaccine, even those who understand its value might be persuaded to opt-out. Vaccination rates increase in many communities when people witness friends, colleagues, and neighbors getting vaccinated without an AEFI. Likewise, poor vaccine uptake might benefit from thwarting the spread of lay epidemiology. Reports suggest that VH can be diminished by citing scientific evidence tailored to the individual patient’s needs and perceptions. Evidence should be presented as images and stories rather than numerical abstractions. Secondly, local leaders and healthcare workers should be involved in addressing issues because they are familiar with local beliefs and the culture of the community. Moreover, healthcare workers should earn trust by demonstrating that effective vaccinations can stop the pandemic.

Systemic AEFIs and the spread of COVID-19 infections among the vaccinated have together weakened people’s trust in vaccines. People wonder why COVID-19 cases do not decline steadily, even with successful global mass vaccination campaigns. We should implement reforms that enhance active immunity, strategically and proactively disseminating the positive aspects of immunization, and persuading subjects to treat AEFIs as a sign of vaccines actually taking effect. Improving trust in government programs can assuage VH in the context of public uncertainty and panic. Together, by considering and addressing the people’s concerns, focus should be on improvising people’s confidence in the vaccine.

Based on our findings, we recommend that the institutions and authorities concerned formulate a policy that restricts the publishing of insignificant AEFIs, especially for ongoing epidemics and pandemics that create panic about impending vaccinations. Because VH can create barriers and impede progress in controlling the spread of the pandemic, we also recommend establishing a centralized national body that scrutinizes all AEFIs reports, conducts casualty assessment and ascertains their authenticity. Publishers will be able to publish these reported AEFIs only after their review and approval. Currently, peer review is not only potentially prejudiced but also rests on the opinion of two or three individuals. On the other hand, formal procedures for scrutinizing, authenticating, and granting prior approval would discourage the reporting of trivial AEFIs and its vigorous spread by social media, aggravating VH calamitously.

### 4.4. Limitations

This narrative review puts forward an opinion that VH is promoted primarily by the indiscriminate dissemination of misleading or false information, including unnecessary and mass AEFI reporting, through the medical literature and social media platforms. Because the reasons for VH are multifactorial and not definable, all contributing reasons should be carefully addressed. While our study suggests that the indiscriminate mass reporting of AEFIs across social media platforms has aggravated COVID-19 VH, broader investigations involving different populations across different countries would be required for creating informed policy.

## 5. Conclusions

VH is a deep-rooted phenomenon, but it is difficult to zero in on the exact reason for it. Spreading rumors/misinformation on COVID-19 vaccines might be an important provocation for VH, which includes reporting AEFI on a massive scale. While a number of reported AEFIs fall within the acceptable limits when huge populations are receiving COVID-19 vaccinations, it is important to critically evaluate and moderate the reporting and dissemination of AEFIs in order to allay panic. Vaccination being the only way to end the COVID-19 pandemic, anything that augments VH must be subject to governmental surveillance. VH during COVID-19 pandemic is a lesson learned, and its prevention would require governmental strategies that restrict random AEFI reports by lay epidemiologists.

## Data Availability

Not applicable.
